# Domain adaptation via Wasserstein distance and discrepancy metric for chest X-ray image classification

**DOI:** 10.1038/s41598-024-53311-w

**Published:** 2024-02-01

**Authors:** Bishi He, Yuanjiao Chen, Darong Zhu, Zhe Xu

**Affiliations:** 1 School of Automation (School of Artificial Intelligence), Hangzhou Dianzi University, Hangzhou, China; 2 Affiliated Hangzhou First People’s Hospital, Zhejiang University School of Medicine, Hangzhou, China

**Keywords:** Medical research, Engineering, Mathematics and computing

## Abstract

Deep learning technology can effectively assist physicians in diagnosing chest radiographs. Conventional domain adaptation methods suffer from inaccurate lesion region localization, large errors in feature extraction, and a large number of model parameters. To address these problems, we propose a novel domain-adaptive method WDDM to achieve abnormal identification of chest radiographic images by combining Wasserstein distance and difference measures. Specifically, our method uses BiFormer as a multi-scale feature extractor to extract deep feature representations of data samples, which focuses more on discriminant features than convolutional neural networks and Swin Transformer. In addition, based on the loss minimization of Wasserstein distance and contrast domain differences, the source domain samples closest to the target domain are selected to achieve similarity and dissimilarity across domains. Experimental results show that compared with the non-transfer method that directly uses the network trained in the source domain to classify the target domain, our method has an average AUC increase of 14.8% and above. In short, our method achieves higher classification accuracy and better generalization performance.

## Introduction

Chest X-ray is one of the most common imaging examination methods, which can be used for early screening of lung inflammation, nodular masses, heart disease, tuberculosis, and other diseases. The imaging physicians can use X-ray images to make a basic diagnosis of the patient’s chest lesion to facilitate further treatment. However, doctors have to interpret millions of reports every year, leading to the existence of missed diagnoses and misdiagnoses, which delay patients’ treatment and increase medical costs.

With the continuous maturation of deep learning techniques and the public availability of many large medical image datasets, convolutional neural networks (CNNs) have made qualitative leaps in image classification and detection. For instance, Wang et al.^1^ evaluated the effectiveness of four classical CNN models, namely AlexNet, GoogleNet, VGGNet-16, and ResNet-50, in classifying chest diseases while compiling and releasing the ChestX-Ray14 dataset. A more famous study is the CheXNeXt model proposed by Rajpurkar et al.^2^, which can simultaneously detect the presence of 14 different pathologies. In diagnosing 11 of these diseases, AI performs comparably to human radiologists. Kumar et al.^3^ then investigated loss functions more suitable for the classification of chest diseases and proposed an enhanced cascade network. Baltruschat et al.^4^ added features such as angle and gender to the model, fully considering the impact of non-image features on disease classification. Shin et al.^5^ proposed a cascaded network that can be used to annotate chest X-rays, providing new ideas for chest radiograph retrieval. Gundel et al.^6^ used multiple datasets (e.g., PLCO dataset and ChestX-Ray14 dataset) for detailed localization labeling and fusion of lung disease to compensate for the lack of information in separate datasets and further improved the diagnosis accuracy of pulmonary diseases.

Transformer was originally proposed by Google and used for natural language processing tasks^7^. Due to the prominent performance of Transformer in handling long sequence data, some researchers started to explore its application to image classification tasks. For example, Dosovitskiy et al.^8^ proposed the Vision Transformer (ViT) model, which splits image data into text sequences similar to those in natural language processing, and then used the Transformer model to model and represent this sequence. This work demonstrates the feasibility of Transformer model in image classification tasks. Later, Liu et al.^9^ proposed Swin Transformer, which divided the input image into small patches by introducing the shifted window mechanism and processing them using a local attention mechanism. Such a mechanism results in a significant reduction in both computational complexity and memory usage, while still maintaining good image classification performance.

Although recent studies have shown good results, it is still difficult to collect fully annotated large-scale chest X-rays as a training set. Besides, the collected training set is supposed to have the same distribution as the test set, because applying the trained model to the test set leads to significant performance degradation when there is a certain difference in data distribution between the trainset and the test set. In addition, due to the lack of labeling of domestic X-ray chest radiograph datasets, the research on chest radiograph lesion detection has been limited to foreign datasets, and the domestic datasets have not played their research value and provided no reliable help for disease diagnosis by imaging doctors in China. Moreover, the labeling of medical images is usually expensive and time-consuming, especially for the study of image data from multiple imaging centers with different machines and equipment, which leads to migration of image distribution due to the differences in scanning protocols, shooting parameters and angles, and subject groups.

Domain adaptation technique is a method to resolve the distribution differences between two data sets and has become a research hotspot in recent years. According to the background setting of the problem, domain adaptation methods can be divided into two categories. The first category is domain adaptation where the label space and feature space of the source and target domains are the same, which is the general case of domain adaptation. The second category is domain adaptation under complex conditions. General domain adaptation methods usually reduce the generalization error of the target domain by reducing the difference between the two domains^10,11^, or by using autoencoders to extract transferable features^12,13^, to ensure that the transfer process does not destroy the original information of the data. Domain adaptation under complex conditions includes multiple sub-directions, such as domain adaptation with inconsistent label spaces and domain adaptation under complex target domain conditions. Cao et al.^14^ use classifiers to output a private label space and a shared label space, so that each target domain sample is only aligned with the most relevant source domain sample, thereby excluding the source domain private categories during the alignment process. Xiao et al.^15^ explore the relationship between different categories through implicit semantics and achieve semantic hierarchy alignment. Gholami et al.^16^ improve the domain separation network to solve the domain adaptation problem of multiple target domains.

The deep domain adaptation methods mentioned above usually outperform traditional algorithms in terms of accuracy but still suffer from some drawbacks in terms of performance and cannot be applied well in practice. Therefore, it is important to design a domain adaptation-based chest radiograph abnormality recognition method that can learn more useful knowledge in the training set (source domain) to migrate to the test set (target domain) and improve the evaluation performance of chest X-ray image classification with fewer parameters. Our main contributions are summarized as follows: (1) We use the hierarchical feature maps constructed by BiFormer to extract deep feature representations in chest radiographs, so as to filter the RoIs (regions of interest) that are most favorable for the chest radiograph classification task. (2) We propose a new domain adaptation method (WDDM) based on the loss minimization of Wasserstein distance and contrast domain differences to achieve similarity and dissimilarity across domains by closing the distance between samples of the same category and pulling apart the distance between different categories in the feature space. Such a method helps to improve the accuracy of chest radiographs classification with better generalization ability. (3) Our research provides a new idea for the domain adaptation task of medical imaging, which has important theoretical and practical significance for promoting the development and application of domain adaptation technology in the field of medical imaging.

## Related work

In recent years, with their superior performance to traditional machine learning methods, deep learning methods have been successfully applied to many fields and have received increasing attention from researchers. Deep neural networks have strong feature extraction ability, which use a multi-layer network structure to obtain higher-level semantic information in data samples. The deep learning method applied to the domain adaptation problem is called deep domain adaptation, whose core idea is to align the data distribution between the source and target domains using deep neural networks. Compared with traditional methods, the features obtained by deep domain adaptation methods not only have stronger generalization ability but also better transferability.

Discrepancy-based domain adaptation methods map features to a high-dimensional RKHS space and use Maximum Mean Discrepancy (MMD)^17^ or similar metrics to measure the discrepancy between two domains. However, traditional metrics such as MMD can usually only detect global discrepancies between domains and have limited effectiveness in detecting local discrepancies. If two domains are similar in most areas but have some local discrepancies, MMD may not be able to accurately detect these discrepancies, which can affect the effectiveness of domain adaptation. In addition, MMD requires a large amount of labeled data to train the model for comparison between different domains. If the amount of labeled data is limited, it may lead to inaccurate estimation of discrepancies. Moreover, MMD often lacks interpretability, i.e., it cannot provide specific information about the discrepancies between domains, making it difficult to determine which features or attributes cause the discrepancies between domains, and thus difficult to further adjust the model and optimize the effectiveness of domain adaptation. Pan et al.^18^ proposed Transferrable Prototypical Networks (TPN), which focuses on the discrepancy between each category in the data set embedding space and assigns pseudo-labels to unlabeled target samples. By adapting the domain, the prototype of each category is made closer in the embedding space. However, the TPN method has limited sensitivity to cross-domain discrepancies. If there are commonalities and discrepancies between two domains, but there is no clear separation in the data set embedding space, TPN may not be able to effectively distinguish these discrepancies, leading to large fluctuations in accuracy. Additionally, TPN relies on the representation of the original data, such as embedding space and prototype, which may result in different sensitivities of the model to different representation methods. If the chosen representation does not fit the sample distribution, it may degrade the performance of the model.

Model-based domain adaptation methods separate the model into several sub-modules and then adjust the parameters of some sub-modules to adapt to domain changes. In this direction, self-training is the most common domain adaptation method, which improves the model in a self-supervised way. Liang et al.^19^ labeled the target samples based on the predictions of the source model and then performed self-supervised learning based on these pseudo-labels. In the process of generating pseudo-labels, the key is how to generate class prototypes and assign pseudo-labels to other samples. Huang et al.^20^ selected samples with self-entropy greater than a certain threshold in each class as class prototypes. However, this method requires manual setting of the self-entropy threshold. If the selected threshold is inappropriate, it will lead to inaccurate class prototype selection. Additionally, for small sample classes, this method cannot select enough prototypes, which can affect the model’s generalization ability. Ding et al.^21^ defined the weight of the source classifier as the class prototype. However, this method ignores the importance of small sample and imbalanced classes and the selected class prototypes are not representative. Xie et al.^22^, inspired by active learning, believed that target samples with higher free energy are more representative of the target domain distribution. Another method for selecting representative class prototypes is to calculate the centroid of each class based on DeepCluster^23^ and assign pseudo-labels based on the distance or similarity between othersamples and the centroid of that class^24,25^. Recent research^26,27^ suggests that using only one prototype cannot fully characterize a class, so multiple prototypes can be generated for each class. However, in the process of assigning pseudo-labels to unlabeled samples, there may be a large number of noisy labels, leading to the model learning incorrect knowledge. Additionally, assigning pseudo-labels to unlabeled data typically relies on the model’s prediction results, which are affected by sample selection bias, reducing the model’s performance. Therefore, Shen et al.^28^ limited the labeling to a subset of the target domain to ensure the accuracy of the model. This approach is called limited label attachment, but it also has some drawbacks. First, the bias in subset selection may lead to incorrect pseudo-labels if the selected subset does not match the distribution of the entire target domain. Second, the size of the subset also affects the effectiveness of the labeling. A subset that is too small can result in low-quality pseudo-labels, while a subset that is too large can increase computational and storage costs. Finally, the subset selection approach results in some unlabeled data being unused, wasting valuable data resources.

Data-based domain adaptation methods can be divided into two categories. The first type of method simulates source domain data or reconstructs an intermediate domain to compensate for missing source domain data when source domain data is unavailable. For example, Liu et al.^29^ proposed a batch normalization statistical loss method based on knowledge distillation without source data^30^, which models the source domain distribution using the mean and variance stored in the BN layer of the source domain model. However, this method is only applicable to source and target domains with similar distributions. Tian et al.^31^ constructed a Gaussian mixture model that implicitly includes prototype information for each class in the weight of the source classifier and derived the mean and standard deviation of the model based on the source classifier. However, the Gaussian mixture model requires prior knowledge of the prototype information for each class and is difficult to apply to multi-label classification problems. Yeh et al.^32^ modeled the inference process and the generation process separately and derived a mixture of Gaussian distributions as a reference distribution from the predicted class. However, using a generative model in a virtual domain is not only expensive but also difficult to achieve domain generalization in the presence of complex data patterns. Therefore, some methods attempt to use non-generative methods, such as directly selecting reliable data from the target domain to construct a virtual source domain. This approach involves feeding target domain images into the source model and representing the source domain distribution with samples with high prediction entropy. However, this method may lead to model overfitting and cannot fully utilize the information from the source domain. The second type of method explores the potential data structure or clustering information in unlabeled target domain data to perform domain adaptation tasks. Yang et al.^33^ proposed using local structural clustering for consistency constraints, which moves feature points from the same cluster to the same category, thereby forming clear clusters in the feature space. Tian et al.^34^ combined pseudo-labeling techniques to obtain structure-preserving pseudo-labels by taking the weighted average prediction of neighboring nodes. However, previous methods only consider maintaining consistency within the same cluster, i.e., reducing intra-class distance, but ignore the differences between different clusters, failing to increase inter-class distance.

The underlying idea behind adversarial domain adaptation methods is the game process between a feature extractor and a domain discriminator. Typical adversarial domain adaptation methods include the multi-adversarial domain adaptation (MADA)^35^, the improved conditional domain adversarial network (CDAN)^36^, the domain adversarial neural network (DANN)^37^, and the adversarial discriminative domain adaptation (ADDA)^38^. Among them, MADA uses the landmark local domain discriminator for taking charge of domain adaptation for each class and optimizes it with conditional probability distributions. The CDAN framework extends the conditional adversarial mechanism to solve the unsupervised domain adaptation problems by defining domain discriminators on features to efficiently align the multi-model distribution of different domains. Such an approach enables differentiated and transferable domain adaptation on class information, and the target performance is improved, but the parameters are also increased substantially. DANN uses feature extractors and domain discriminators for adversarial training, where the domain discriminators are global domain discriminators that do not distinguish classes and can be said to optimize the edge probability distribution. However, during the convergence of the validation process, this adversarial training approach makes the feature extractor in the target domain focus not only on RoIs but also on background regions, leading to a larger error in extracting features. In contrast, ADDA uses two feature extraction networks acting on the source and target domains to facilitate different optimization operations on data from different domains, but its adversarial training process makes the feature extraction network in the target domain to focus on the background region, leading to the inability to correctly locate the lesion regions. The latest attempt to improve adversarial discriminative models is Smooth Domain Adversarial Training (SDAT)^39^, which achieves smooth minimization of task-specific losses and helps better adapt to the target domain. However, this method requires additional smoothing of input data and the model may focus too much on smoothness and ignore other important features.

Recent research shows that CNN is not necessary for image classification tasks, and good performance can also be achieved by applying Transformer directly to a sequence of image patches. In traditional CNNs, convolution and pooling operations are widely used for image feature extraction and dimensionality reduction. In contrast, the multi-head self-attention mechanism used in Transformer can also achieve similar feature extraction and fusion effects, and thus play an important role in image classification tasks. For instance, Touvron et al.^40^ proposed the DeiT model to train ViT using a knowledge distillation strategy to achieve competitive performance with less pre-trained data. Wang et al.^41^ proposed the PVT model to port Transformer to various dense prediction tasks, which not only allowed training on dense partitions of images to obtain high output resolution but also used progressive shrinkage pyramids to reduce the computation of large feature maps. Dai et al.^42^ proposed CoatNet, which took full advantage of CNN and self-attention mechanism to design a new transformer module to focus on both local and global information. Zhu et al.^43^ proposed a Transformer network architecture based on dynamic sparse attention to alleviate the scalability issue of multi-head self-attention.

A brief overview of the development of domain adaptation networks and the characteristics of different methods. Sun et al.^44^ proposed Unsupervised Domain Adaptation (UDA), which aligns the learned representations of the source and target domains using self-supervised auxiliary tasks. Compared to DANN, UDA replaces the domain classification task with a rotation and flipping task. Compared to DANN, ADDA uses two feature extraction networks acting on the source and target domains respectively, instead of using only one feature extraction network. It can be seen that the main change of these domain adaptive networks is not in the network structure but in the feature extraction method. BiFormer has a layered structure similar to CNN and is capable of learning multi-scale features, which can be easily applied to downstream tasks. In this paper, we take BiFormer, which replaces the traditional CNN to perform deep feature extraction on data samples. Moreover, we propose a novel loss minimization strategy based on Wasserstein distance and contrast domain differences to evaluate the chest radiograph classification effect.

## Methods

**Figure 1:**
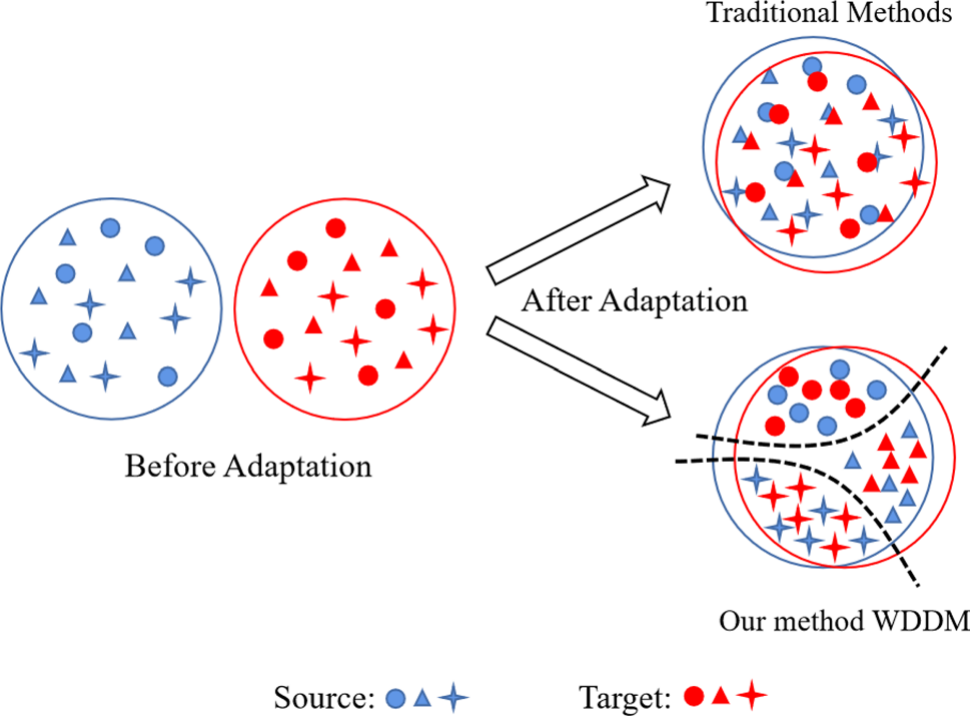
Comparison between the traditional domain adaption methods and our proposed WDDM method.

This paper presents a domain adaptation method called the Joint Wasserstein Distance and Discrepancy Metric (WDDM) for abnormality recognition in chest X-ray images. The proposed method aims to overcome the limitations of traditional domain adaptation methods, such as inaccurate lesion region localization, large feature extraction errors, and high model parameter complexity. In our method, we assume that the same category labels exist in the source and target domains, and our method does not need to rely on the label information of the target domain data when performing tasks, which enables us to handle the situation where the target domain lacks accurate labels. Although previous domain adaptation methods have shown good performance on many public datasets, there are still some limitations in aligning the source and target domains. As shown in Fig. 1, traditional domain adaptation methods simply close the distance between the source and target domains without considering the category labels of the samples and only achieve domain-level alignment, which may lead to poor classification results in multiple classification scenarios. Wasserstein distance is one of the traditional domain adaptation methods. It only reduces the domain offset between the source domain and the target domain and does not achieve alignment between categories. To address this problem, based on Wasserstein distance aligned domain distribution, we use the category labels of each sample to calculate the distribution difference between the two domains and make the samples of the same category closer and the samples of different categories gradually separated. WDDM can be regarded as a fine-grained alignment process on top of the traditional domain adaptation methods.

**Figure 2:**
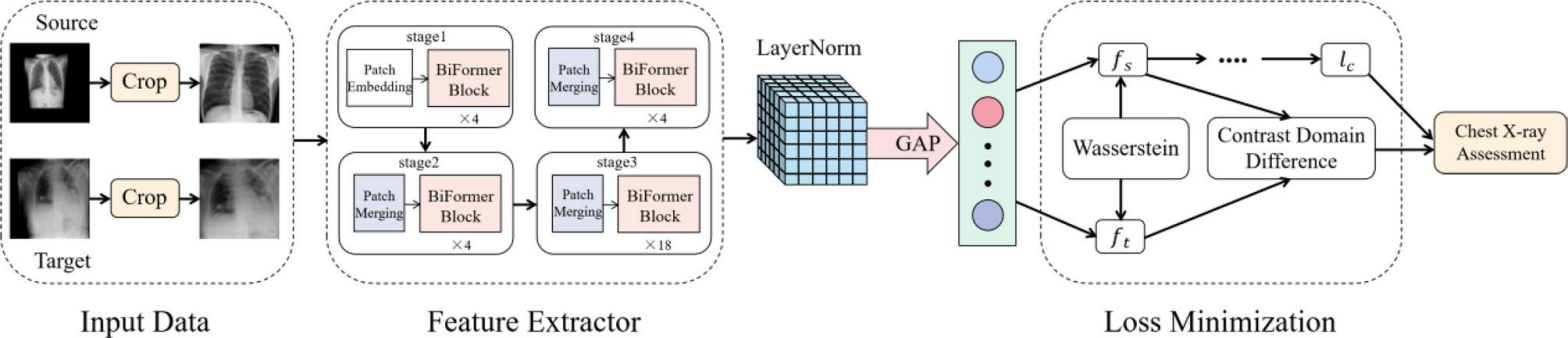
The general framework diagram of WDDM. ${f}_{s}$ and ${f}_{t}$ represent the feature vectors in the source and target domains, respectively. ${l}_{c}$ is the cross-entropy loss obtained from the source domain training.

Figure 2 illustrates the general framework of WDDM. Specifically, our framework consists of four parts. The first part is the input module, which is used to obtain images of chest radiographs and perform data preprocessing operations on them. The second part is the feature extraction module, and BiFormer is selected as the feature extractor. First, we determine the number and the size of patches. Then, the feature maps of different sizes are constructed sequentially through four stages. As the network deepens, the number of patches decreases and the perceptual range of each patch expands, which facilitates the layer construction of BiFormer. The third part is the loss minimization module. First, the Wasserstein distance between the source domain samples and the target domain samples is calculated and the closest two domain samples are selected. Then, the similarity and dissimilarity of the two domain samples are sparsely processed across domains using the contrast domain difference. Finally, the total objective function is constructed to realize the optimization and parameter update of BiFormer network. The fourth part is the validation module, which is used to validate the optimized and parameter-updated BiFormer network and perform the classification prediction task of chest radiographs.

For the input module in the first part, after obtaining the chest X-ray image, we perform preprocessing operations on it in terms of standardization and data enhancement. First, use the mean and standard deviation of the image to normalize so that the data distribution meets the standard normal distribution, and then enhance the data through cropping and random rotation. See “Data pre-processing module” section for details. In the verification module of the fourth part, we use the trained model to classify and predict the images in the target domain test set, using six methods: AUC, Accuracy, Sensitivity, Specificity, Positive Predictive Value (PPV), and Negative Predictive Value (NPV). Performance evaluation indicators are used to evaluate the classification effect of the model. Next, we will introduce the second and third parts in the WDDM framework in detail.

### Multi-scale feature extraction based on BiFormer

**Figure 3:**
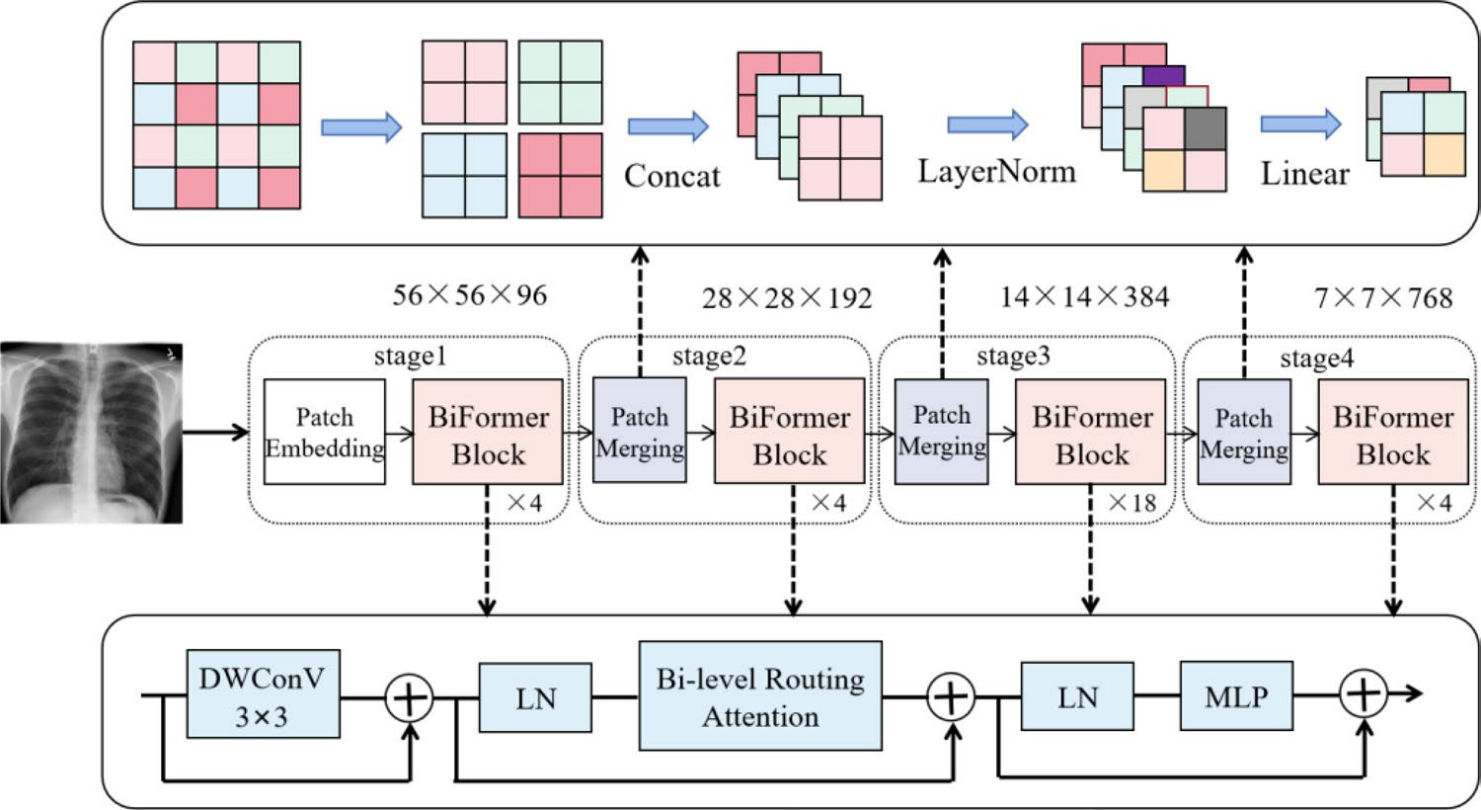
Schematic diagram of feature extraction network.

Unlike the previous domain adaptive methods, BiFormer is selected as the multi-scale feature extractor in this paper, and the schematic diagram of the feature extraction network is shown in Fig. 3. BiFormer utilizes patch merging operation, similar to pooling, to synthesizes four adjacent small patches into one large patch. Then, the pixels in the same position of each patch are stitched together to form four feature maps, which are concatenated in the depth direction. After Patch Merging, the height and width of the feature map are halved, while the depth is doubled, thereby increasing the receptive field of each convolutional kernel and generating multi-scale features.

The pre-processed chest images are fed into the feature extractor. The input chest radiograph is a three-channel image whose height ${\text{H}}$ and width ${\text{W}}$ of the image are set to 224 × 224. After downsampling 32 times in four stages of BiFormer, the output feature matrix is of size 7 × 7. Firstly, the image is input to the Patch Partition module for chunking, setting every 4 × 4 = 16 adjacent pixels as a patch, and each pixel has three values of R, G, and B. The shape of the image is changed from (32, 3, 224, 224) to (32, 48, 56, 56) after spreading in the channel direction, and the channel data of each pixel is linearly transformed by the linear embedding layer. Then, the BiFormer blocks are stacked repeatedly in four stages to construct feature maps of different sizes.

The number of blocks stacked in the four stages of BiFormer-B model is (4, 4, 18, 4). Specifically, in the first stage, the channel data for each pixel is linearly transformed via a linear embedding layer to embed the features into 96 dimensions, and the shape of the image changes from (32, 48, 56, 56) to (32, 96, 56, 56). The next three stages all downsample by a factor of two based on the output of the previous stage, with the image shape changes as (32, 96, 56, 56) → (32, 192, 28, 28) → (32, 384, 14, 14) → (32, 768, 7, 7). In this way, a multi-level feature map is generated to make the characterization of chest radiographs more recognizable. The structural configuration of the four stages of BiFormer-B is shown in Table 1.

**Table 1:** Structural configuration for the four stages of the BiFormer-B model.

	Stage1	Stage2	Stage3	Stage4
Channel depth of feature map	96	192	384	768
Number of blocks stacked	4	4	18	4

**Figure 4:**
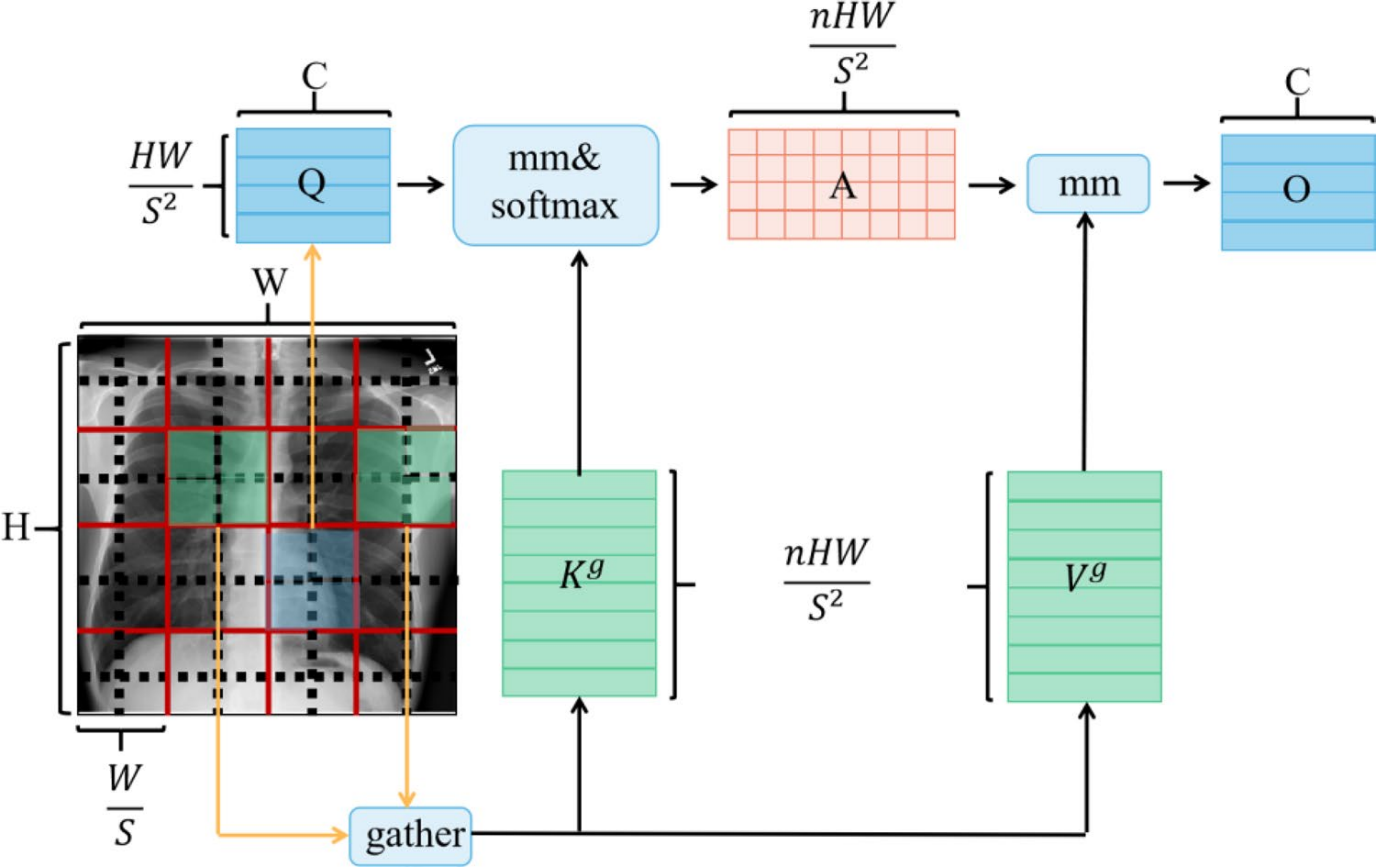
Schematic diagram of the Bi-level Routing Attention module, where ${\text{H}}$ represents the height of the feature map, W represents the width, ${\text{C}}$ represents the depth, ${\text{O}}$ represents the complexity, ${\text{S}}$ represents the square root of the number of regions, ${\text{A}}$ represents the adjacency matrix, ${\text{n}}$ represents the number of regions to participate, ${\text{Q}}$ represents the query tensor, ${\text{K}}$ represents the key tensor, ${\text{V}}$ represents the value tensor, and ${\text{mm}}$ represents matrix multiplication.

In the process of stacking BiFormer Blocks, a 3 × 3 depthwise convolution (DWconv) is first used to implicitly encode relative positional information. Then, the Bi-level Routing Attention module and Multi-Layer Perceptron (MLP) module are applied in sequence for cross-position relationship modeling and position embedding, respectively. The Bi-level Routing Attention module, shown in Fig. 4, is used to filter out most of the irrelevant key-value pairs at the coarse area level, retaining only a small number of routing areas. This removes redundant information, achieving more flexible calculation allocation and higher classification performance.

For the input chest X-ray feature map, QKV is obtained through linear mapping. Then, a directed graph is constructed using the adjacency matrix to find the participating relationships for different key-value pairs, i.e., the regions that each given region should participate in. Once the routing index matrix from region to region is obtained, fine-grained token-to-token attention can be applied. From Fig. 4, it can be seen that the Bi-level Routing Attention module collects key-value pairs from the top k related windows and utilizes sparsity operations to skip the calculation of the least relevant areas, thereby saving parameter and computation costs. In addition, since BiFormer focuses on a small subset of relevant tokens in an adaptive query manner rather than dispersing attention to other irrelevant tokens, it has good performance and high computational efficiency.

For the chest radiograph classification task, the BiFormer network is followed by a Layer Norm layer, a global pooling layer, and a fully connected layer to obtain the final output.

### Loss minimization based on Wasserstein distance and contrast domain difference

The loss function of our model consists of three components, which are the cross-entropy loss of the source domain training, the Wasserstein distance of the source domain samples closest to the target domain samples, and the contrast domain difference of similar similarity and dissimilarity.

Define the source domain dataset ${{\text{D}}}_{{\text{S}}}$ obey the probability distribution ${{{\text{D}}}_{{\text{S}}}\sim {\text{X}}}_{{\text{S}}}$ and the target domain dataset ${{\text{D}}}_{{\text{T}}}$ obey the probability distribution ${{{\text{D}}}_{{\text{T}}}\sim {\text{X}}}_{{\text{T}}}$. As shown in the Loss Minimization part of the network framework in Fig. 2, the feature vectors of the source domain and the target domain are obtained from the output of the fully connected layer, and the distribution difference between the source domain and the target domain is measured by calculating the Wasserstein distance between them. Compared with KL divergence and JS divergence, one advantage of Wasserstein distance is that it can reflect the distance between the two domains even when there is little overlap in the sample distributions. If there is no overlap or negligible overlap between the two distributions, KL divergence may be meaningless and JS divergence is fixed as a constant, which means the gradient is 0 for the gradient descent method. However, Wasserstein distance is smooth and it can provide a more stable gradient. Wasserstein distance is defined as

1 $$ W(X_{S} ,X_{T} ) = \mathop {\inf }\limits_{{\gamma \sim \prod (X_{S} ,X_{T} )}} E_{(p,q)\sim \gamma } \left[ {\left\| {p - q} \right\|} \right], $$

where $\prod ({{\text{X}}}_{{\text{S}}},{{\text{X}}}_{{\text{T}}})$ denotes the set of all possible joint distributions of the source domain distribution ${{\text{X}}}_{{\text{S}}}$ and the target domain distribution ${{\text{X}}}_{{\text{T}}}$. $\upgamma \sim \prod ({{\text{X}}}_{{\text{S}}},{{\text{X}}}_{{\text{T}}})$ depicts the set of distributions from ${{\text{X}}}_{{\text{S}}}$ shifting to ${{\text{X}}}_{{\text{T}}}$ and thus the cost required to make both obey the same distribution. For each of the possible joint distributions $\upgamma$ can be sampled from $({\text{p}},{\text{q}})\sim\upgamma$ get a sample of ${\text{p}}$ and ${\text{q}}$ and calculate the sample ${\text{p}}$ and ${\text{q}}$ the distance between $\Vert {\text{p}}-{\text{q}}\Vert$, one obtains the expected value of the distance under this joint distribution $\upgamma$, the expectation value of the sample to the distance ${{\text{E}}}_{({\text{p}},{\text{q}})\sim\upgamma }[\Vert {\text{p}}-{\text{q}}\Vert ]$. The smaller the expected value of ${{\text{X}}}_{{\text{S}}}$, the smaller the expected value of ${{\text{X}}}_{{\text{T}}}$. The process of obtaining the infimum on the expectation of the sample-to-distance is the process of selecting the source domain sample that is closest to the target domain sample.

Since it is not easy to find the infimum, the Wasserstein distance can be written in the form of the dual based on the Kantorovich–Rubenstein duality, as shown in Eq. (2).

2 $$ W(X_{S} ,X_{T} ) = \mathop {\sup }\limits_{{\left\| g \right\|_{L} \le 1}} E_{{D_{S} \sim X_{S} }} \left[ {g(D_{S} )} \right] - E_{{D_{T} \sim X_{T} }} \left[ {g(D_{T} )} \right], $$

where ${\text{sup}}$ denotes the supremum, that is, the maximum value of the expected difference is obtained for all functions ${\text{g}}({\text{x}})$ that satisfy the conditions. ${{\text{E}}}_{{{\text{D}}}_{{\text{S}}}\sim {{\text{X}}}_{{\text{S}}}}[{\text{g}}({{\text{D}}}_{{\text{S}}})]$ and ${{\text{E}}}_{{{\text{D}}}_{{\text{T}}}\sim {{\text{X}}}_{{\text{T}}}}[{\text{g}}({{\text{D}}}_{{\text{T}}})]$ respectively represent the expected value of the source domain samples ${{\text{D}}}_{{\text{S}}}$ and the target domain samples ${{\text{D}}}_{{\text{T}}}$ for the function ${\text{g}}({\text{x}})$ under the marginal probability distributions ${{\text{X}}}_{{\text{S}}}$ and ${{\text{X}}}_{{\text{T}}}.$ The significance of the dual form is that a Lipschitz continuous function ${\text{g}}({\text{x}})$ can be found through network iterative optimization. Under the condition that the Lipschitz constant does not exceed 1, the expectation of the source domain distribution and the target domain distribution on ${\text{g}}({\text{x}})$ is calculated, such that ${{\text{E}}}_{{{\text{D}}}_{{\text{S}}}\sim {{\text{X}}}_{{\text{S}}}}\left[{\text{g}}\left({{\text{D}}}_{{\text{S}}}\right)\right]-{{\text{E}}}_{{{\text{D}}}_{{\text{T}}}\sim {{\text{X}}}_{{\text{T}}}}[{\text{g}}({{\text{D}}}_{{\text{T}}})]$ is maximum, used to estimate the Wasserstein distance between two distributions. ${\Vert g\Vert }_{L}$ denotes the Lipschitz function, which is defined as

3 $$ \left\| g \right\|_{L} = \sup \left| {g(x_{1} ) - g(x_{2} )} \right|/\left| {x_{1} - x_{2} } \right|. $$

Contrast domain difference is then used to perform category label-level alignment for domain adaptation, and intra- and inter-class differences are jointly optimized to improve adaptive performance. The underlying labeling assumptions of the target domain samples are estimated by the K-means clustering method. The time complexity of the K-means clustering algorithm is linear, the convergence speed is fast, and it is relatively scalable and efficient in processing large data sets. The Hierarchical Clustering algorithm has great demands on time and space. The difficulty lies in the selection of merging or splitting points. If the merging or splitting points are not well selected at a certain step, it may lead to low-quality clustering results, and This clustering method does not scale well. It is difficult for the DBSCAN clustering algorithm to find appropriate density parameters for cluster structures with greatly different densities, and the time complexity of the algorithm is high. The convergence speed of the Gaussian Mixture Model is slow and it is easy to converge to the local optimum. Therefore, compared with other clustering algorithms, K-means clustering algorithm can deal with large data sets more effectively, converge to stable clustering results in a short time, and achieve a better balance between speed and scalability. In addition, the core idea of introducing contrast domain differences in the clustering process is to minimize the differences between the same categories and maximize the differences between different categories. Then the feature representation of intra-class samples can be compressed based on the contrast domain difference, and the feature representation of inter-class samples can be further pushed away from the decision boundary, as shown in Eq. (4).

4 $$ D^{cdd} = \frac{1}{N}\sum\limits_{c = 0}^{N - 1} {D^{cc} (y_{{1:n_{t} }}^{t} ,\sigma )} - \frac{1}{N(N - 1)}\sum\limits_{c = 1}^{N - 1} {\sum\limits_{c^{\prime} = 1,c^{\prime} \ne c}^{N - 1} {D^{cc^{\prime}} (y_{{1:n_{t} }}^{t} ,\sigma )} }, $$

where ${\text{N}}$ is the number of classification categories, ${n}_{t}$ is the size of the target domain dataset. ${y}_{1:{n}_{t}}^{t}=\{{y}_{1}^{t},{y}_{2}^{t},...,{y}_{{n}_{t}}^{t}\}$ represents the set of category labels in the target domain, and ${{\text{D}}}^{\mathrm{cc^{\prime}}}$ is the average embedding estimation of category ${\text{c}}$ and category $\mathrm{c^{\prime}}$ in the regenerative kernel Hilbert space. It is worth noting that $\upsigma$ is the feature representation extracted from the network through the labeled target sample and labeled source sample provided in the clustering stage. According to the current feature representation, the label of the target domain is updated through the clustering process. For the data distribution of all categories in the two domains, minimize the contrast domain difference ${D}^{cdd}$ so that the first half of the formula is as small as possible and the second half is as large as possible. That is, for the five lesion types in the chest radiograph, the ${D}^{cdd}$ loss makes the sample distribution of the same category close and pull the sample distribution of different category away in the source and target domain.

After clustering, the fuzzy target domain data far from the cluster center and fuzzy classes containing few target samples around the cluster center are set to zero when estimating the contrast domain difference. And the model becomes more accurate as more classes are involved as the network continues to be trained.

Finally, the Wasserstein distance and contrast domain difference obtained above are introduced into the classification network as the regular terms of the loss function, resulting in the overall objective function

5 $$ \mathop {\min }\limits_{\theta } l = l_{c} + \lambda W(X_{S} ,X_{T} ) + \eta D^{cdd}, $$

where ${l}_{c}$ is the cross-entropy loss obtained in the source domain training process, $\uplambda$ and $\upeta$ are the weights to balance the losses of Wasserstein distance and contrast domain difference, respectively. We use the overall loss to update the model parameters until the model converges.

### Network optimization and hyperparameters setting

The network is optimized by adjusting the feature representation through backpropagation. During the training iterations, to improve training efficiency, class-aware sampling is used for the source and target domains, i.e., data is sampled from both domains for each class within a randomly sampled subset of classes. First, the network parameters are fixed and the sample clusters in the target domain are updated. Then, each target domain sample is given a label corresponding to the belonging cluster, and the updated target domain label is used to calculate the contrast domain difference. The Wasserstein distance between the two domain samples is calculated to determine the data samples to participate in the contrast domain difference calculation. Finally, the network parameters are updated by minimizing the contrast domain difference until the iteration is completed.

**Figure 5:**
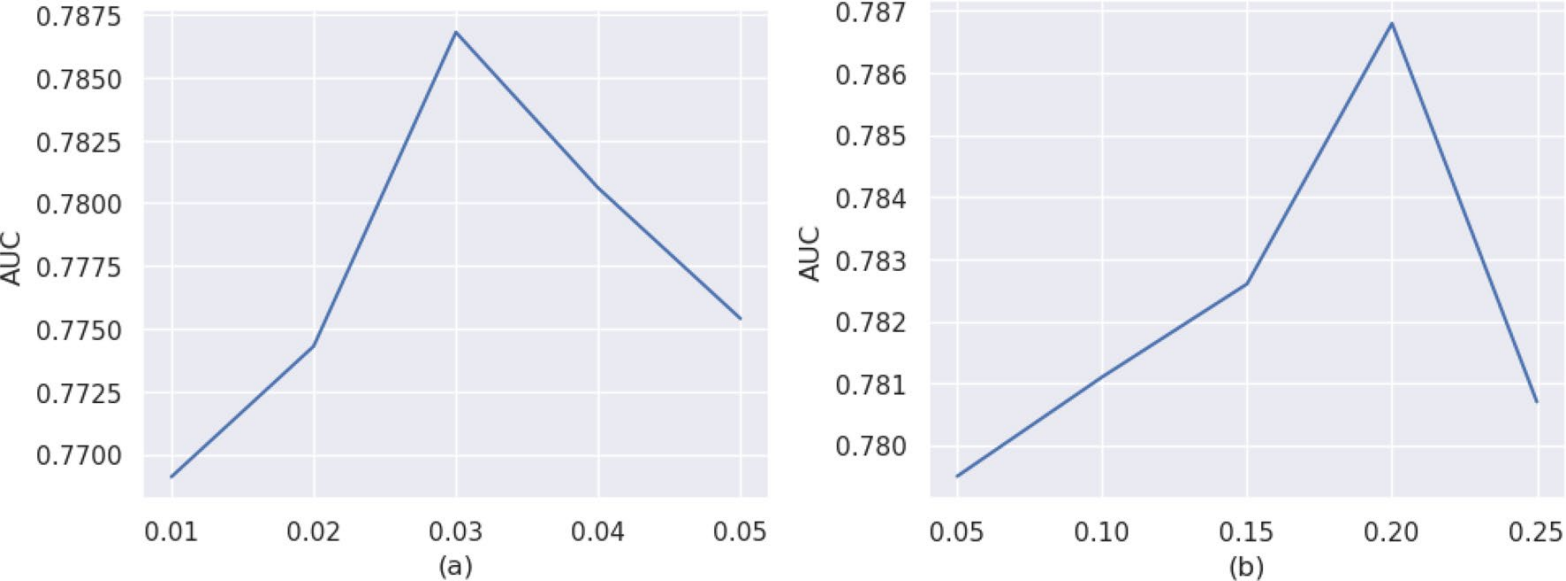
Sensitivity study of hyperparameter, (**a**) AUC values under different $\uplambda$, (**b**) AUC values under different $\upeta$.

In addition, we investigate the impact of the weights $\uplambda$ and $\upeta$ on the performance. $\uplambda$ and $\upeta$ control the importance of the Wasserstein distance and the contrast domain difference, which make the calculation of the difference measure more accurate. Therefore, we adjust the loss function by adjusting $\uplambda$ and $\upeta$ values to investigate their sensitivities and the results are shown in Fig. 5.

As can be seen in Fig. 4a, when $\uplambda$ increases from 0.01 to 0.03, the AUC value increases, while when $\uplambda$ is greater than 0.03, the AUC value starts to gradually decrease. As shown in Fig. 4b, the AUC reaches the maximum value when $\uplambda$ = 0.20. Therefore, we set $\uplambda$ = 0.03 and $\upeta$ = 0.2.

## Experiments and results

### Data set and evaluation indicators

A total of three chest radiograph datasets are used in this paper: CheXpert, Chest X-Ray14, and PadChest. The CheXpert dataset contains 224,316 radiographic images of 65,240 patients taken from the front, back, and side, with most of the images being frontal or lateral views. The dataset includes uncertain medical labels and reference standard evaluation sets annotated by radiologists, which can be used to predict the probabilities of 14 different observations for multi-view chest X-ray images. The Chest X-Ray14 dataset contains 112,120 frontal chest X-ray images from 30,805 patients, and the radiology reports include 14 common diseases. The PadChest dataset contains 160,868 images from 67,625 patients, with 19 types of labels.

**Figure 6:**
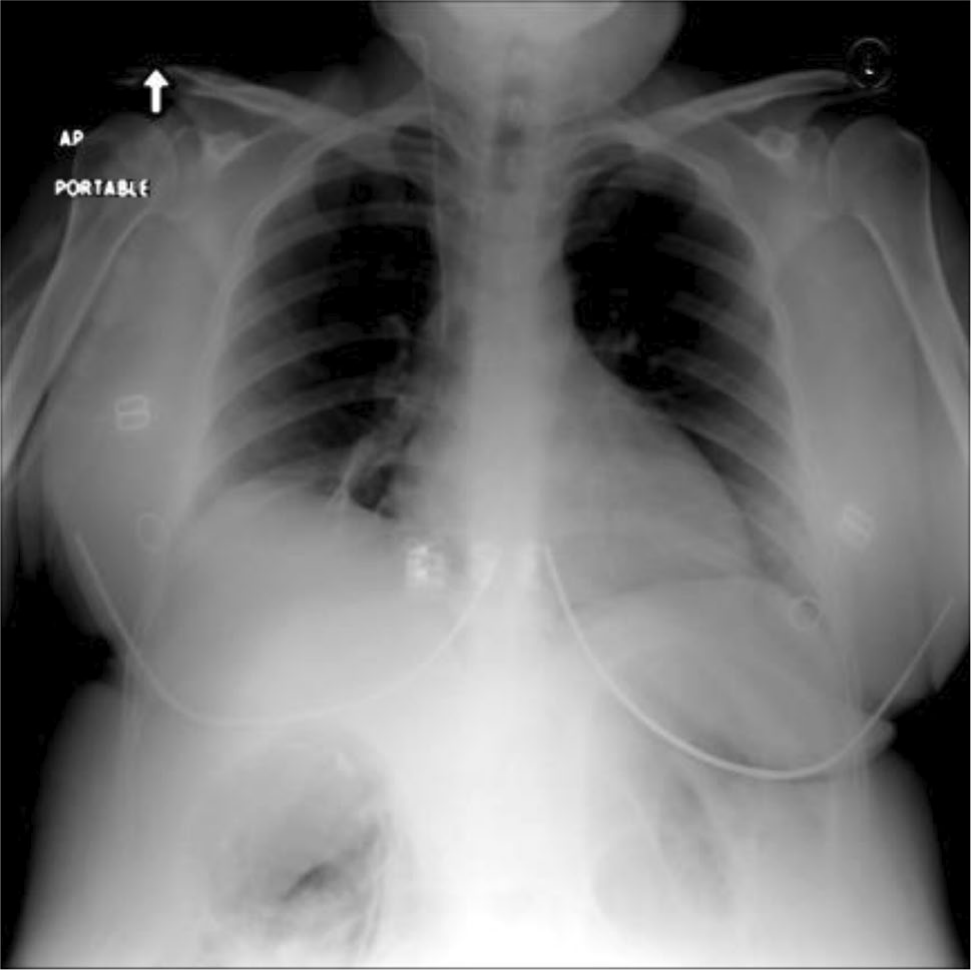
Example of a chest radiograph with the corresponding label 01110, where ‘0’ means no such lesion and ‘1’ means the presence of such lesion, indicating that this chest radiograph contains three types of lesions: Cardiomegaly, Effusion, and Consolidation.

The labels of the three data sets do not completely overlap. In our study, five lesion types that are present in all three datasets are selected, which are Atelectasis, Cardiomegaly, Effusion, Consolidation, and Edema. A single chest radiograph may contain multiple lesion types, as shown in Fig. 6.

**Figure 7:**
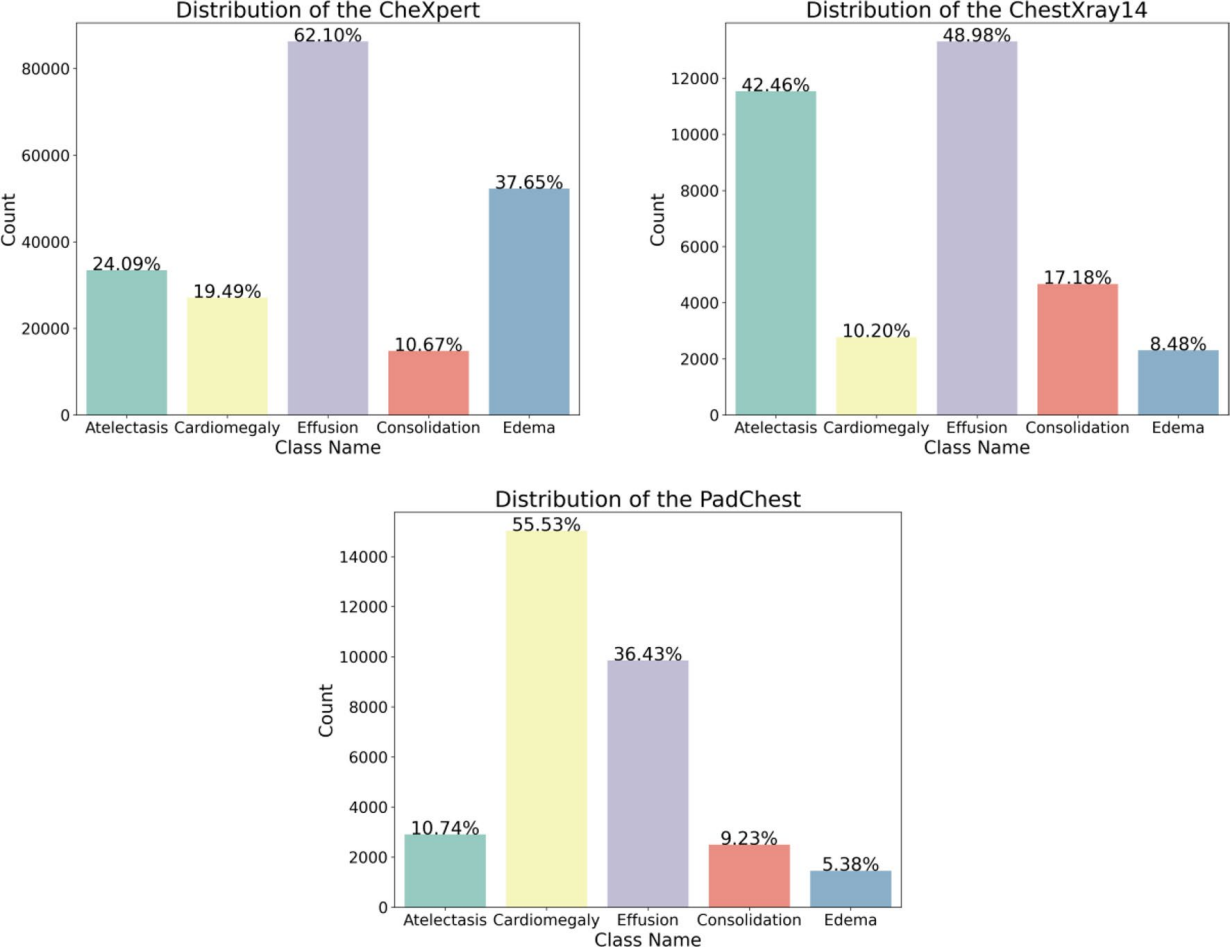
Percentage distribution of the five lesion types in the CheXpert, ChestXray14, and PadChest datasets.

After excluding the chest radiographs that did not contain these five types of lesions, 138,894 samples remained in the CheXpert dataset, of which 24.09% are Atelectasis, 19.49% are Cardiomegaly, 62.10% are Effusion, 10.67% are Consolidation, and 37.65% are Edema. 27,167 samples remained in the Chest X-ray14 dataset, of which 42.46% are Atelectasis, 10.20% are Cardiomegaly, 48.98% are Effusion, 17.18% are Consolidation, and 8.48% are Edema. 27,043 samples remained in the PadChest dataset, of which 10.74% are Atelectasis, 55.53% are Cardiomegaly, 36.43% are Effusion, 9.23% are Consolidation, and 5.38% are Edema. The distributions of lesions in each dataset are shown in Fig. 7.

We performed fivefold cross-validation on the CheXpert dataset and the ChestX-Ray14 dataset based on the proportions of the five lesion types. For the PadChest data set, it is randomly divided according to the ratio of 8:1:1. Among them, 22,050 images are used for training, 2500 images are used for testing, and 2500 images are used for verification. The three data sets are randomly divided into training set, test set and validation set. Keep the proportion of lesion types in the training set, test set, and validation set close to ensure a balanced distribution of abnormal data.

In the verification mode, the predicted labels are used to calculate the confusion matrix TP/TN/FP/FN, as well as the AUC, Accuracy, Sensitivity, Specificity, Positive Prediction Value (PPV), and Negative Prediction Value (NPV) of each category of chest radiographs in the test set. Evaluate the classification performance of the model. The AUC is the area under the ROC curve, and the closer the value is to 1, the better the classification performance of the model. The horizontal coordinate of the ROC curve is the False Positive Rate (FPR), which is calculated as

6 $$ FPR = FP/(FP + TN). $$

The vertical coordinate of the ROC curve is True Positive Rate (TPR), which is calculated as

7 $$ TPR = TP/(TP + FN). $$

The accuracy rate is calculated as

8 $$ Accuracy = (TP + TN)/(TP + TN + FP + FN). $$

The formula for calculating the sensitivity is shown in Eq. (9).

9 $$ {\text{S}}ensitivity = TP/(TP + FN). $$

The formula for calculating the specificity is shown in Eq. (10).

10 $$ Specificity = TN/(TN + FP). $$

The positive predictive value was calculated as shown in Eq. (11).

11 $$ PPV = TP/(TP + FP). $$

The negative predictive value was calculated as shown in Eq. (12).

12 $$ NPV = TN/(TN + FN). $$

where TP denotes positive samples predicted as positive category, TN denotes negative samples predicted as negative category, FP denotes negative samples predicted as positive category, and FN denotes positive samples predicted as negative category. In the case of multiple classification, the positive category corresponds to a certain category of lesion ${\text{c}}$ and the negative class corresponds to other categories $\mathrm{c^{\prime}}$ of lesions.

### Data pre-processing module

The data preprocessing module includes two steps: first, normalization is performed using the mean and standard deviation of the images and the images are uniformly scaled to (32, 3, 224, 224). Then, the data are enhanced by cropping and random rotation. According to the distribution of image regions, the images are cropped and randomly rotated with an offset of no more than ± 25°, without affecting the possible abnormal regions.

### Implementation details

The source domain datasets for this experiment are the CheXpert dataset and PadChest dataset, and the target domain is the Chest X-Ray14 dataset. To make the comparison results more informative, we conducted thirteen comparison experiments. In addition, in order to ensure that the model does not cause errors due to the division of target instances into training sets and test sets, we use fivefold cross-validation for the CheXpert data set and Chest X-Ray14 data set. The target domain data set is randomly divided into 5 parts, one part is randomly selected as the test set, and the remaining 4 parts are integrated with the source domain instances as the training set, and 5 sets of experiments are conducted in sequence.

Experiment 1 is set as a dual source domain (CheXpert and PadChest datasets) and a single target domain (Chest X-Ray14 dataset) to demonstrate that the classification accuracy is not necessarily improved by the increased number of chest radiographs. The CheXpert dataset and PadChest dataset are first merged as the source domain to train the classifier and save the parameters of the best model, and then the target domain Chest X-Ray14 dataset is directly used to classify the chest X-ray abnormalities. That is, experiment 1 does not use any domain adaptation method to close the distance between the source and target domains. Experiments 2 to 13 are performed for single source domain and single target domain, which are CheXpert → Chest X-Ray14, CheXpert → PadChest, Chest X-Ray14 → CheXpert, Chest X-Ray14 → PadChest, PadChest → Chest X-Ray14, and PadChest → CheXpert, respectively. To demonstrate the effectiveness of WDDM for improving the generalization ability of the chest radiograph classification model, we train experiments 2 to 13 twice: once with WDDM and once without the domain adaptation method. In addition, the network model chosen for the experiments without using the domain adaptive method is the Swin Transformer.

During the training of the above thirteen experiments, we chose Adam as the optimizer with a momentum of 0.9. The initial learning rate is 0.0001, and the decay rate is 0.0001. In addition, we trained a total of 100 rounds with a batch size of 16.

### Experimental results and performance analysis

**Figure 8:**
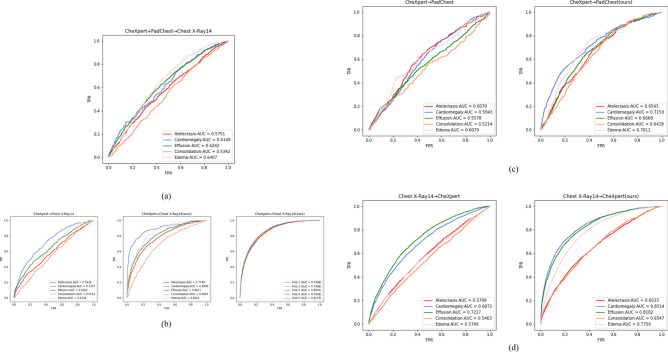

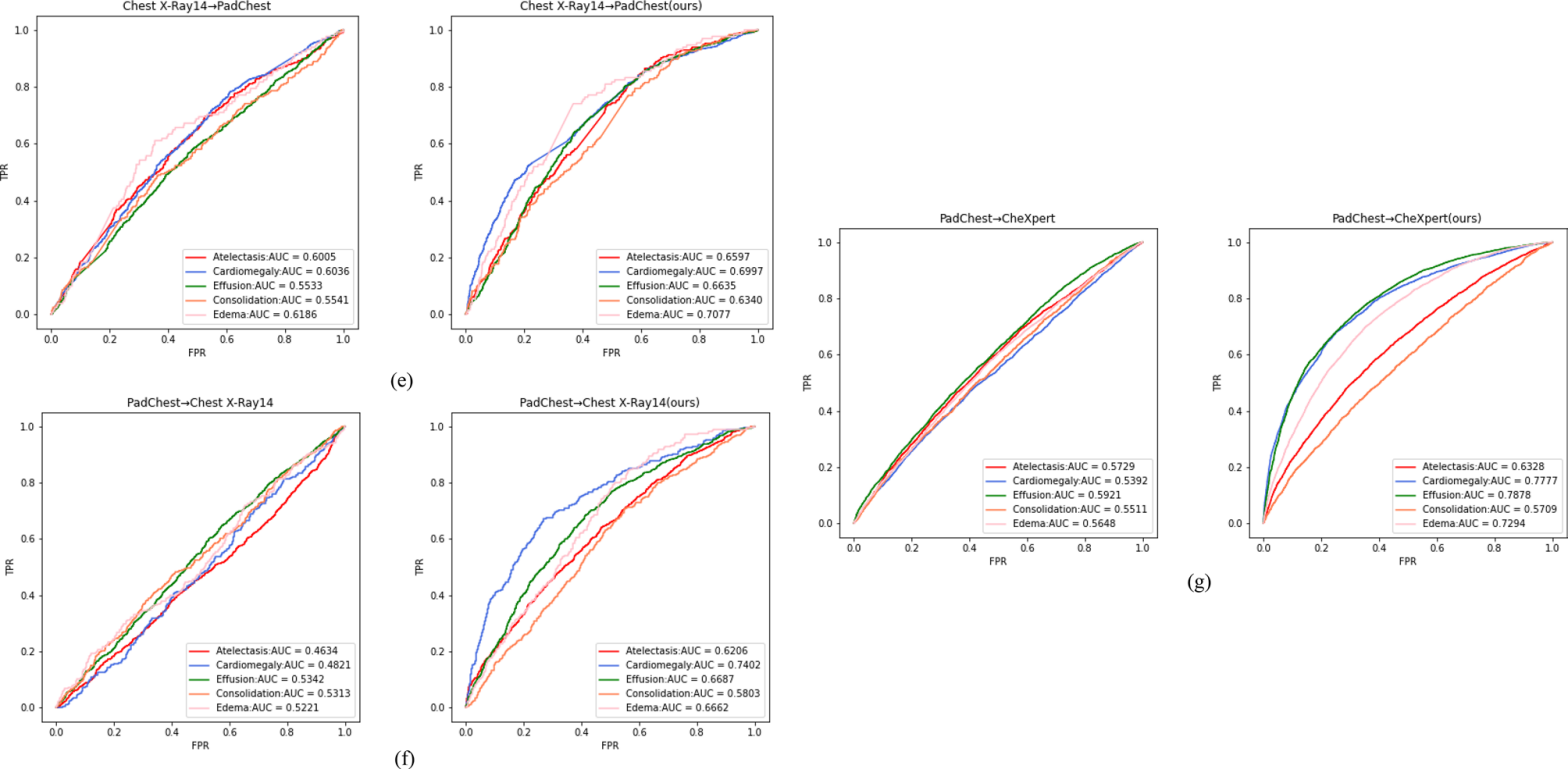
The ROC curves of thirteen experiments on the target domain test set. Figure (**a**) shows the ROC curves without the domain adaptive method in the dual source domain and single target domain, and Figures (**b–g**) show the ROC curves of the method without domain adaptive and our method in the single source domain and single target domain. For Figure (**b**), we used the WDDM method to conduct a fivefold cross-validation experiment on the CheXpert data set and the Chest X-Ray14 data set.

The AUC value of the model on the validation set is calculated for each epoch, and the performance of the validation set is used to determine whether the current training model is the best. After training, the parameters of best model are used to perform classification prediction tasks. Multiple evaluation metrics are calculated to evaluate the classification effect. Figure 8 shows the ROC curves obtained for thirteen experiments on the target domain test set, and Table 2 compares the classification evaluation metrics of the thirteen experiments.

**Table 2:** Classification evaluation metrics (%) for thirteen experiments.

		AUC	Accuracy	Sensitivity	Specificity	PPV	NPV
Exp 1	CheXpert + PadChest → Chest X-Ray14	59.8	55.5	60.2	54.8	30.0	79.3
Exp 2	CheXpert → Chest X-Ray14	62.7	58.7	58.4	59.9	31.5	79.6
Exp 3	CheXpert → Chest X-Ray14(Ours)	**80.5**	**74.3**	**73.8**	**74.4**	**45.3**	**87.2**
Exp 4	CheXpert → PadChest	57.8	57.3	58.4	56.5	27.8	80.5
Exp 5	CheXpert → PadChest(Ours)	**67.6**	**61.7**	**67.5**	**60.3**	**31.7**	**84.4**
Exp 6	Chest X-Ray14 → CheXpert	62.2	59.2	58.9	59.2	37.8	76.3
Exp 7	Chest X-Ray14 → CheXpert(Ours)	**74.7**	**69.0**	**67.6**	**69.5**	**46.9**	**82.5**
Exp 8	Chest X-Ray14 → PadChest	58.6	59.4	56.0	60.0	28.4	80.0
Exp 9	Chest X-Ray14 → PadChest(Ours)	**67.3**	**60.7**	**64.6**	**62.7**	**33.0**	**83.2**
Exp 10	PadChest → Chest X-Ray14	50.7	49.6	53.6	48.8	26.3	75.5
Exp 11	PadChest → Chest X-Ray14(Ours)	**65.5**	**60.6**	**66.2**	**58.9**	**33.6**	**81.9**
Exp 12	PadChest → CheXpert	56.4	55.5	54.0	55.6	34.4	72.9
Exp 13	PadChest → CheXpert(Ours)	**70.0**	**65.3**	**64.7**	**65.4**	**43.8**	**80.6**

Significant values are in bold.

From Fig. 8, we can observe how the classification performance of each experiment changes under different thresholds. The closer the ROC curve is to the upper left corner, the better the performance of the model in the target domain. The curves in Figures (b) to (g) provide a visual comparison between the model improved using the WDDM method and the model without the domain adaptation method under different experimental settings. By comparing the shape and position of the curves, we can evaluate the effectiveness of our proposed method for different experiments. It can be observed that the classification performance on the target domain is improved in experiments using the WDDM method compared to experiments not using the domain adaptation method. The curve is closer to the upper left corner, indicating that the WDDM method can effectively reduce the differences between domains and improve the classification accuracy in the target domain. In addition, through cross-validation experiments on the CheXpert dataset and Chest X-Ray14 dataset, the results further support the reliability and validity of the WDDM method used in our study.

Table 2 lists the classification evaluation indicators of 13 experiments, including AUC, Accuracy, Sensitivity, Specificity, PPV and NPV. The source domain and target domain data sets of each experiment are also explained in the table. Comparing the classification evaluation metrics of each experiment, it can be seen that training a classifier using the combined CheXpert and PadChest X-ray chest imaging datasets as the source domain and directly testing on Chest X-Ray14 resulted in an average AUC of 0.598 for the five disease categories. In contrast, testing CheXpert alone on Chest X-Ray14 resulted in an average AUC of 0.627, and testing PadChest alone on Chest X-Ray14 resulted in an average AUC of 0.507. This suggests that simply merging multiple source domains into the training dataset may not improve the disease classification performance for chest radiographs, as the data distributions in different source domains may not be entirely similar and domain shift may exist between source domains. This result supports the viewpoint of Luo et al.^45^. In addition, the CheXpert data set is a larger-scale chest X-ray data set, which provides more image samples, allowing the trained model to have stronger generalization capabilities in the target domain. In contrast, the Chest X-Ray14 dataset and PadChest dataset have relatively weak generalization capabilities due to small sample sizes or limitations in specific fields. This may affect its classification performance on the target domain, making the model perform worse than the model trained on the CheXpert dataset when facing new unseen samples. Therefore, when designing experiments and interpreting results, we also considered the impact of the size and diversity of the data set on the generalization ability of the model.

As seen in Fig. 8 and Table 2, our method achieves better performance in several chest radiograph datasets, indicating the generalization and applicability of WDDM, which benefits from its unique design. We use the multi-scale feature extraction module of BiFormer to classify features at different scales. This module extracts features at different scales to obtain richer and more representative feature representations, which improves the robustness and generalizability of the model. and makes it more generalizable. Based on the loss minimization of Wasserstein distance and contrast domain difference, we effectively reduce the domain differences between source and target domains to help the model better adapt to different domain data and improve domain adaptation effects. Extensive experiments show that WDDM achieves better results in various evaluation metrics, not only in terms of AUC value but also in accuracy, precision, specificity, positive predictive value, negative predictive value, and other metrics, demonstrating an overall improvement in model performance. In addition, WDDM can distinguish samples from two different domains more accurately and effectively avoids the performance degradation problem caused by distribution differences between domains by adaptive learning. Moreover, the model is iterated and updated during the training process to maintain the accuracy and generalization ability, which is beneficial for practical applications. It should also be noted, that the unbalanced sample size of different lesions results in lower positive predictive values for certain types of lesions, as shown in Table 3.

**Table 3:** Positive predictive value for five types of lesions in the test set from CheXpert → Chest X-Ray14 using WDDM.

Type	Atelectasis	Cardiomegaly	Effusion	Consolidation	Edema
PPV	0.6614	0.3108	0.7025	0.2943	0.2334

Table 3 demonstrates the positive predictive values of five types of lesions on the Chest X-Ray14 test set by CheXpert → Chest X-Ray14 using WDDM. Since the samples with Edema has the smallest proportion on the Chest X-Ray14 dataset, the positive predictive value for this type of lesion is lower than the remaining four types of lesions. More accurate labeling can improve the learning effect of the model, mitigate the impact of unbalanced sample distribution, and improve the prediction ability of the model in real scenarios.

Similarly, the CheXpert dataset is better classified as a source domain than the PadChest or Chest X-Ray14 dataset because the CheXpert dataset has more samples and contains more information than the PadChest and Chest X-Ray14 datasets. Overall, the comparative experiments on various single-source and single-target domains indicate that WDDM has significant advantages in performance and practicality.

### Ablation studies

#### Domain adaptation methods

**Figure 9:**
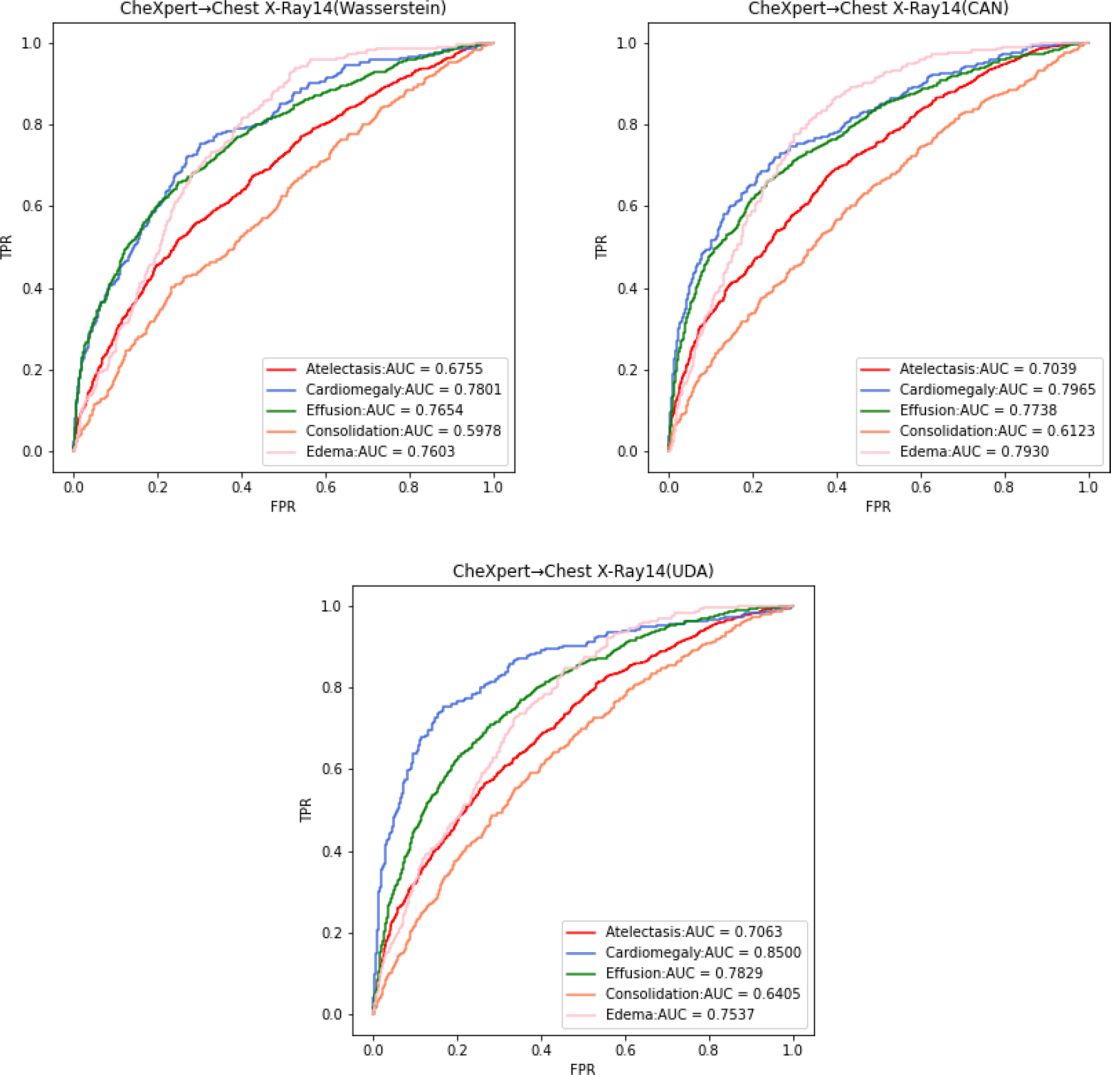
ROC curves for Wasserstein distance, CAN, and UDA on the test set.

We choose the experiment without domain adaptation methods as the base model and conduct comparative experiments on four different domain adaptation methods using the CheXpert dataset as the source domain and the Chest X-Ray14 dataset as the target domain. The first domain adaptation method is Wasserstein distance, which calculates the Wasserstein distance between the source and target domain feature vectors as a part of the total loss to update model parameters until it converges. This method helps the deep network to learn the source domain classification task while continuously reducing the domain offset, so that the deep network model gradually transitions from the classification task acting on the source domain to the target domain, which efficiently improves the classification performance on the target domain. The second domain adaptation method is Contrastive Adaptation Network (CAN)^46^, which adds category labels as the condition for computing the difference. The core process of CAN is to improve the domain adaptive performance of the target domain by calculating the MMD with a separate category of data for the source and target domains, minimizing this difference if they are two identical classes, and maximizing this difference otherwise. The third domain adaptation method is UDA, which induces alignment between the source and target domains by learning self-supervised auxiliary tasks in both domains. The fourth domain adaptation method is WDDM proposed by us. Figure 9 shows the ROC curves of Wasserstein distance, CAN, and UDA on the Chest X-Ray14 test set.

As can be seen in Fig. 9, the domain adaptation method using Wasserstein distance achieves an average AUC of 0.7158 on the test set for the five categories of lesions, indicating that the model reduces the domain shift between the source and target domains by optimizing the Wasserstein distance loss during training. The domain adaptation method using CAN obtains an even higher average AUC of 0.7359, because it considers the distribution distance between different categories and is more stable in the presence of label noise when dealing with large amounts of data. The domain adaptation method using UDA had an average AUC of 0.7467. Each self-supervised task aligned the two domains in the direction of the variation relevant to that task, and all self-supervised tasks on both domains were trained together with the original task on the source domain, resulting in representations that were well aligned. Our method WDDM achieves an average AUC of 0.8046 on the test set, demonstrating its effectiveness in cross-domain medical image classification tasks. Compared with other methods, WDDM achieved a higher AUC value, indicating that our model has better prediction ability, higher accuracy, and reliability in solving chest X-ray domain adaptation problems. Therefore, our method has better application prospects. In order to describe the behavior and performance of the algorithms in the experimental part more clearly, we provide in Table 4 the technical composition of the five models and a detailed comparison of their AUC values and Accuracy on CheXpert → Chest X-Ray14.

**Table 4:** Technical comparison and experimental results of different methods (%).

Methods	BiFormer-backbone	Category alignment	AUC	Accuracy
Base	No	No	62.7	58.7
Wasserstein distance	No	No	71.6	66.8
CAN	No	Yes	73.6	67.7
UDA	No	No	74.7	69.6
WDDM (ours)	Yes	Yes	**80.5**	**74.3**

Significant values are in bold.

As shown in Table 4, the base model has the lowest mean AUC value of 0.627 for its five lesion categories, indicating that there is indeed a domain offset between the source and target domains, resulting in poor classification. In contrast, WDDM has the best classification effect on the target domain test set, with a mean AUC value of 28.4% higher than that of the one-to-one base model without the domain adaptation method, 12.4% higher than that of the method with Wasserstein distance, 9.4% higher than that of the method with CAN, and 7.8% higher than that of the method with UDA. Therefore, WDDM has greater advantages in the acquisition of key information from chest radiographs, and can achieve better prediction performance and generalization performance. The ablation study fully demonstrates the feasibility and effectiveness of WDDM.

#### Model selection

In BiFormer, there are three model architectures: BiFormer-T, BiFormer-S, and BiFormer-B, with^2,2,2,8^ block stacking numbers for BiFormer-T and^4,4,4,18^ for both BiFormer-S and BiFormer-B. In Swin Transformer, there are four model architectures: Swin-T, Swin-S, Swin-B, and Swin-L. We conducted comparative experiments on ResNet50, two model architectures from Swin Transformer (Swin-S and Swin-B), and two model architectures from BiFormer (BiFormer-S and BiFormer-B). Table 5 compares the parameter amounts, FLOPs of each backbone network, and their AUC values on the Chest X-Ray14 test set.

**Table 5:** Comparison of different backbone networks.

Models	Parameters (M)	FLOPs (G)	AUC (%)
ResNet50	26	4.1	61.1
Swin-S	50	8.7	76.9
Swin-B	88	15.4	77.3
BiFormer-S	26	4.5	78.6
BiFormer-B	57	9.8	**80.5**

Significant values are in bold.

From Table 5, we can observe that the ResNet50 model has a smaller number of parameters and a lower amount of calculation in the target task, but its AUC value is also the lowest. This shows that ResNet50 is relatively lightweight, but slightly insufficient in classification performance. In contrast, the Swin-S and Swin-B models significantly outperform ResNet50 in the chest X-ray image classification task by increasing the model size and calculation amount. The BiFormer-S and BiFormer-B models are lower than Swin-S and Swin-B in terms of number of parameters and calculations, but their AUC values on the Chest X-Ray14 test set are higher than those of ResNet50 and Swin Transformer, indicating that BiFormer A better speed-accuracy trade-off is achieved. Additionally, the BiFormer-B model achieves the highest AUC value of 0.8050. Compared with the other four models, the BiFormer-B model can extract rich features at different levels through its unique design and deeper structure, which results in powerful layer-by-layer feature learning and representation capability. The deeper the network, the more abstract the features are and the more semantic information is extracted, thus improving the performance. It shows that BiFormer has the potential to become an effective model selection. These findings are of great significance to the research and application of medical image classification tasks, and provide a valuable reference for further optimization and improvement of model selection.

### Visualization and interpretation

#### Feature activation heatmap

**Figure 10:**
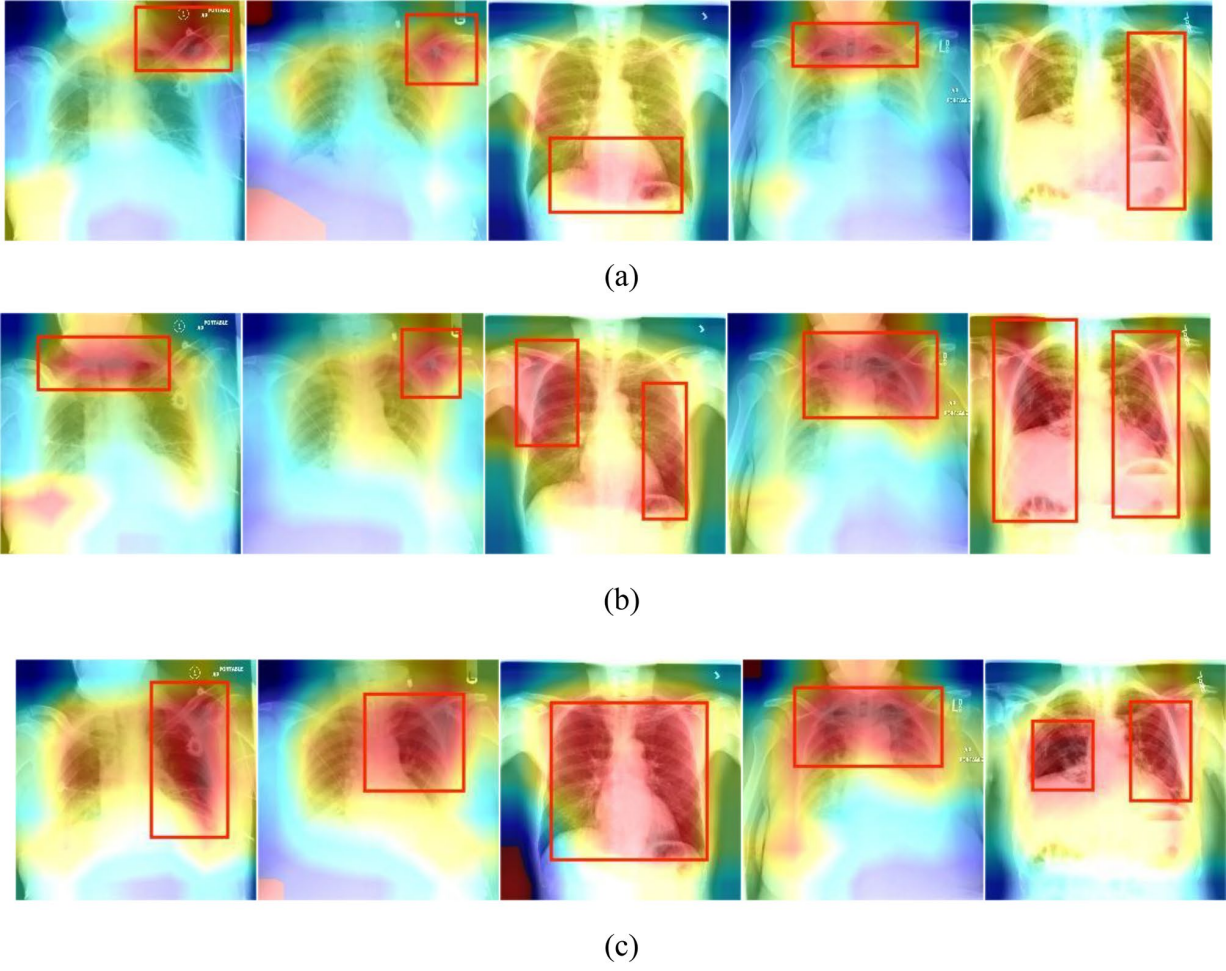
Figure (**a–c**) depict a set of heatmaps produced by ResNet50, Swin Transformer, and BiFormer, respectively.

A set of heat map pairs generated by ResNet50, Swin Transformer and BiFormer on chest radiographs are shown in Fig. 10. The darker the color in the heat map indicates that the model pays more attention to the information in this region, and then that region has a greater impact on the final classification task.

As shown in Fig. 10, in a set of heat maps generated by the ResNet50 model, the model may focus on some areas that do not have actual lesion information in the image. These areas may contain some mediocre features, or the impact of noise on the model, indicating that the ResNet50 model is not robust. In contrast, the Swin Transformer model has better performance when displaying RoIs areas because it changes the feature image size by fusing patches to achieve different levels of attention calculations and has better feature extraction capabilities. Compared with convolutional neural networks and Swin Transformer, BiFormer focuses on a small number of relevant tokens in a query-adaptive manner, and it can filter out most of the feature information irrelevant to the lesion features at the rough area level. In other words, BiFormer pays more attention to the RoIs area where the lesions occur on the chest X-ray, so that the model can focus more on the feature information that is beneficial to the classification task. In addition, based on the loss minimization of Wasserstein distance and contrast domain difference, fuzzy target domain data far from the cluster center and fuzzy classes containing few target samples around the cluster center are set to zero, resulting in the cluster center being surrounded by classes with more similar target samples. Therefore, the differences between individual classes become more and more prominent and the model obtains better classification accuracy.

### Research limitations and future research directions

The quality of chest radiographs varies greatly among different hospitals and devices, which can have an impact on the performance and robustness of the domain adaption algorithm. Therefore, future research focuses on designing more robust and reliable chest radiograph domain adaptive algorithms, and quantifying and analyzing the robustness of the algorithm.

In addition, the experimental results show that the merged chest radiograph datasets had a negative impact on the classification effect, indicating that it is necessary to reduce the differences between individual source domains in multi-source domain adaptation studies. The method described in this paper provides an idea for multi-source domain adaptation research to a certain extent, because the purpose of domain adaptation is to align the target domain with the source domain regardless of whether it is a single source domain or multiple source domains, and the domain adaptation algorithm for single source domains can be applied to multi-source domain adaptation as well. Multi-source domains contain richer potential information than single-source domain, and similar features often exist between multiple source domains, and the rich information can be complemented to each other. How to share knowledge among different source domains and efficiently use this information to apply on the target domain to improve the domain adaptive performance is the key issue that will continue to be studied in the future.

## Conclusion

In this paper, we propose a domain adaptive approach for chest radiograph abnormality identification by joint Wasserstein distance and discrepancy metric, referred to as WDDM. Specifically, first, the BiFormer network is used to obtain deeper feature representation of data samples and capture more useful information. Then, the closest two-domain samples are selected using the Wasserstein distance, and the comparative domain differences are used to close the distance between two domains for the same category and pull apart the distance between different categories to realize similarity and dissimilarity across domains. Numerous experiments have illustrated the effectiveness of WDDM in improving the classification accuracy for chest radiograph abnormality identification, and ablation studies demonstrate the impact of each component of our model on experimental results. In addition, our research can assist some relatively inexperienced imaging physicians in diagnosing diseases on chest radiographs, helping them to discover lesions that are difficult to identify with the naked eye, reducing the incidence of false-negative diagnosis and improving the accuracy of diagnosis, as well as improving the efficiency of radiograph reading, so that physicians can devote their time to more valuable work.

## Data Availability

The datasets generated and/or analysed during the current study are available in the following public repositories: CheXpert (https://stanfordmlgroup.github.io/competitions/chexpert/); PadChest (https://github.com/auriml/Rx-thorax-automatic-captioning); Chest X-Ray14 (https://www.kaggle.com/datasets/nih-chest-xrays/data).

## References

[1] Wang, X. *et al*. Chestx-ray8: Hospital-scale chest X-ray database and benchmarks on weakly-supervised classification and localization of common thorax diseases. In *Proc. IEEE Conference on Computer Vision and Pattern Recognition* 2097–2106 (2017).

[2] Rajpurkar P, Irvin J, Ball RL (2018). Deep learning for chest radiograph diagnosis: A retrospective comparison of the CheXNeXt algorithm to practicing radiologists. PLoS Med..

[3] Kumar, P., Grewal, M. & Srivastava, M. M. Boosted cascaded convnets for multilabel classification of thoracic diseases in chest radiographs. In *Image Analysis and Recognition: 15th International Conference, ICIAR 2018, Póvoa de Varzim, Portugal, June 27–29, 2018, Proceedings 15* 546–552 (Springer, 2018).

[4] Baltruschat IM, Nickisch H, Grass M (2019). Comparison of deep learning approaches for multi-label chest X-ray classification. Sci. Rep..

[5] Shin, H. C. *et al*. Learning to read chest X-rays: Recurrent neural cascade model for automated image annotation. In *Proc. IEEE Conference on Computer Vision and Pattern Recognition* 2497–2506 (2016).

[6] Guendel, S. *et al*. Learning to recognize abnormalities in chest X-rays with location-aware dense networks. In *Progress in Pattern Recognition, Image Analysis, Computer Vision, and Applications: 23rd Iberoamerican Congress, CIARP 2018, Madrid, Spain, November 19–22, 2018, Proceedings 23* 757–765 (Springer, 2019).

[7] Vaswani A, Shazeer N, Parmar N (2017). Attention is all you need. Adv. Neural Inf. Process. Syst..

[8] Dosovitskiy, A. *et al*. An image is worth 16x16 words: Transformers for image recognition at scale. Preprint at http://arXiv.org/2010.11929 (2020).

[9] Liu, Z. *et al*. Swin transformer: Hierarchical vision transformer using shifted windows. In *Proc. IEEE/CVF International Conference on Computer Vision* 10012–10022 (2021).

[10] Chen C, Chen Z, Jiang B (2019). Joint domain alignment and discriminative feature learning for unsupervised deep domain adaptation. Proc. AAAI Conf. Artif. Intell..

[11] Lee, C. Y. *et al*. Sliced wasserstein discrepancy for unsupervised domain adaptation. In *Proc. IEEE/CVF Conference on Computer Vision and Pattern Recognition* 10285–10295 (2019).

[12] Sun, R. *et al*. Not all areas are equal: Transfer learning for semantic segmentation via hierarchical region selection. In *Proc. IEEE/CVF Conference on Computer Vision and Pattern Recognition* 4360–4369 (2019).

[13] Zhu, P., Wang, H. & Saligrama, V. Learning classifiers for target domain with limited or no labels. In *International Conference on Machine Learning* 7643–7653 (PMLR, 2019).

[14] Cao, Z. *et al*. Partial adversarial domain adaptation. In *Proc. European Conference on Computer Vision (ECCV)* 135–150 (2018).

[15] Xiao W, Ding Z, Liu H (2021). Implicit semantic response alignment for partial domain adaptation. Adv. Neural Inf. Process. Syst..

[16] Gholami B, Sahu P, Rudovic O (2020). Unsupervised multi-target domain adaptation: An information theoretic approach. IEEE Trans. Image Process..

[17] Borgwardt KM, Gretton A, Rasch MJ (2006). Integrating structured biological data by kernel maximum mean discrepancy. Bioinformatics.

[18] Pan, Y. *et al*. Transferrable prototypical networks for unsupervised domain adaptation. In *Proc. IEEE/CVF Conference on Computer Vision and Pattern Recognition* 2239–2247 (2019).

[19] Liang, J., Hu, D. & Feng, J. Do we really need to access the source data? Source hypothesis transfer for unsupervised domain adaptation. In *International Conference on Machine Learning* 6028–6039 (PMLR, 2020).

[20] Huang, Y. *et al*. Relative alignment network for source-free multimodal video domain adaptation. In *Proc. 30th ACM International Conference on Multimedia* 1652–1660 (2018).

[21] Ding, Y. *et al*. ProxyMix: Proxy-based mixup training with label refinery for source-free domain adaptation. Preprint at http://arXiv.org/2205.14566 (2018). 10.1016/j.neunet.2023.08.005 37634264

[22] Xie B (2022). Active learning for domain adaptation: An energy-based approach. Proc. AAAI Conf. Artif. Intell..

[23] Caron, M. *et al*. Deep clustering for unsupervised learning of visual features. In *Proc. European Conference on Computer Vision (ECCV)* 132–149 (2018).

[24] Wang R (2022). Cross-domain contrastive learning for unsupervised domain adaptation. IEEE Trans. Multimedia.

[25] Qu, S. *et al*. BMD: A general class-balanced multicentric dynamic prototype strategy for source-free domain adaptation. In *Computer Vision–ECCV 2022: 17th European Conference, Tel Aviv, Israel, October 23–27, 2022, Proceedings, Part XXXIV* 165–182 (Springer, 2022).

[26] Ding, Y. *et al*. ProxyMix: Proxy-based mixup training with label refinery for source-free domain adaptation. Preprint at http://arXiv.org/2205.14566 (2022). 10.1016/j.neunet.2023.08.005 37634264

[27] Ahmed, W., Morerio, P. & Murino, V. Cleaning noisy labels by negative ensemble learning for source-free unsupervised domain adaptation. In *Proc. IEEE/CVF Winter Conference on Applications of Computer Vision* 1616–1625 (2022).

[28] Shen, M., Bu, Y. & Wornell, G. On the benefits of selectivity in pseudo-labeling for unsupervised multi-source-free domain adaptation. Preprint at http://arXiv.org/2202.00796 (2022).

[29] Liu, Y., Zhang, W. & Wang, J. Source-free domain adaptation for semantic segmentation. In *Proc. IEEE/CVF Conference on Computer Vision and Pattern Recognition* 1215–1224 (2021).

[30] Yin, H. *et al*. Dreaming to distill: Data-free knowledge transfer via deepinversion. In *Proc. IEEE/CVF Conference on Computer Vision and Pattern Recognition* 8715–8724 (2020).

[31] Tian J, Zhang J, Li W (2021). VDM-DA: Virtual domain modeling for source data-free domain adaptation. IEEE Trans. Circuits Syst. Video Technol..

[32] Yeh, H. W. *et al*. Sofa: Source-data-free feature alignment for unsupervised domain adaptation. In *Proc. IEEE/CVF Winter Conference on Applications of Computer Vision* 474–483 (2021).

[33] Yang, S. *et al*. Generalized source-free domain adaptation. In *Proc. IEEE/CVF International Conference on Computer Vision* 8978–8987 (2021).

[34] Tian L, Zhou L, Zhang H (2023). Robust self-supervised learning for source-free domain adaptation. Signal Image Video Process..

[35] Pei, Z. *et al*. Multi-adversarial domain adaptation. In *Proc. AAAI Conference on Artificial Intelligence*, Vol. 32, 1 (2018).

[36] Long M, Cao Z, Wang J (2018). Conditional adversarial domain adaptation. Adv. Neural Inf. Process. Syst..

[37] Ganin Y, Ustinova E, Ajakan H (2016). Domain-adversarial training of neural networks. J. Mach. Learn. Res..

[38] Tzeng, E. *et al*. Adversarial discriminative domain adaptation. In *Proc. IEEE Conference on Computer Vision and Pattern Recognition* 7167–7176 (2017).

[39] Rangwani, H. *et al*. A closer look at smoothness in domain adversarial training. In *International Conference on Machine Learning* 18378–18399 (PMLR, 2022).

[40] Touvron, H. *et al*. Training data-efficient image transformers & distillation through attention. In *International Conference on Machine Learning* 10347–10357 (PMLR, 2021).

[41] Wang, W. *et al*. Pyramid vision transformer: A versatile backbone for dense prediction without convolutions. In *Proc. IEEE/CVF International Conference on Computer Vision* 568–578 (2021).

[42] Dai Z, Liu H, Le QV (2021). Coatnet: Marrying convolution and attention for all data sizes. Adv. Neural Inf. Process. Syst..

[43] Zhu, L. *et al*. BiFormer: Vision transformer with bi-level routing attention. Preprint at http://arXiv.org/2303.08810 (2023).

[44] Sun, Y. *et al*. Unsupervised domain adaptation through self-supervision. Preprint at http://arXiv.org/1909.11825 (2019).

[45] Luo L, Chen H, Xiao Y (2022). Rethinking annotation granularity for overcoming shortcuts in deep learning-based radiograph diagnosis: A multicenter study. Radiol. Artif. Intell..

[46] Kang, G. *et al*. Contrastive adaptation network for unsupervised domain adaptation. In *2019 IEEE/CVF Conference on Computer Vision and Pattern Recognition (CVPR)* (IEEE, 2020).

